# Networked Estimation for Event-Based Sampling Systems with Packet Dropouts

**DOI:** 10.3390/s90403078

**Published:** 2009-04-24

**Authors:** Vinh Hao Nguyen, Young Soo Suh

**Affiliations:** Department of Electrical Engineering, University of Ulsan, Namgu, Ulsan 680-749, Korea; E-Mail: vinhhao@hcmut.edu.vn (V.H.N.)

**Keywords:** Networked estimation, event-based sampling, send-on-delta, packet dropout

## Abstract

This paper is concerned with a networked estimation problem in which sensor data are transmitted over the network. In the event-based sampling scheme known as level-crossing or send-on-delta (SOD), sensor data are transmitted to the estimator node if the difference between the current sensor value and the last transmitted one is greater than a given threshold. Event-based sampling has been shown to be more efficient than the time-triggered one in some situations, especially in network bandwidth improvement. However, it cannot detect packet dropout situations because data transmission and reception do not use a periodical time-stamp mechanism as found in time-triggered sampling systems. Motivated by this issue, we propose a modified event-based sampling scheme called modified SOD in which sensor data are sent when either the change of sensor output exceeds a given threshold or the time elapses more than a given interval. Through simulation results, we show that the proposed modified SOD sampling significantly improves estimation performance when packet dropouts happen.

## 1. Introduction

Recent works have discussed event-driven alternatives to traditional time-triggered sampling schemes. It has been shown to be more efficient than time-triggered one in some situations, especially in network bandwidth improvement. In [1–7], event-based sampling scheme was applied by adjusting the threshold value at each sensor node, data transmission rate is reduced so that the network can be used for other traffic.

However, analysis and simulation in the the works on event-driven sampling scheme were performed under ideal communication network conditions: no delays or packet dropouts are assumed, but in realistic applications, network induced delays and packet losses do happen.

The issues of network delays and packet dropouts in time-triggered systems have been addressed and solved by researchers in [8–14]. In [8] the stability of the Kalman filter in relation to the data arrival rate is investigated. It is shown that there exists a critical data arrival rate for an unstable system so that the mean filtering error covariance will be bounded for any initial condition. In a very recent study [13], the optimal H_2_ filtering problems associated respectively with possible delay of one sampling period, uncertain observations and multiple packet dropouts are studied under a unified framework. The H_2_-norm of systems with stochastic parameters is defined and computed via a Lyapunov equation and a steady-state filter is designed via an LMI approach. In [14], the authors adopt a model similar to that of [13] for multiple packet dropouts to investigate finite-horizon optimal linear filtering, prediction and smoothing problems.

In conventional event-based sampling systems, also called send-on-delta (SOD) sampling [5–7], the issues of network delay and packet loss are difficult to solve because data transmission and reception do not use a periodical time-stamp mechanism as in the time-triggered sampling systems. Motivated by those issues, in this paper, we introduce a modified SOD sampling scheme in which the event-driven sampling is combined with a time-triggered sampling scheme to detect packet dropouts. Then, a networked estimator based on a Kalman filter is formulated to estimate states of the system periodically even when the sensor nodes do not transmit data. The proposed SOD sampling scheme has properties inherited from the conventional SOD sampling: so the benefits from event-driven sampling are still hold. Through theoretical analysis and simulation results, we show that the proposed SOD sampling scheme gives better estimation performance than the conventional SOD one when packet loss happens.

## 2. Modified SOD Sampling Scheme

Consider a networked control system described by the linear continuous-time model:

(1) $$\begin{array}{l} \dot{x}(t)=\mathrm{Ax}(t)+\mathrm{Bu}(t)+w(t)\\ y(t)=\mathrm{Cx}(t)+v(t) \end{array}$$

where *x*(*t*) ∈ *R^n^* is the state of the plant, *u* is the deterministic input signal, *y*(*t*) ∈ *R^p^* is the measurement output which is sent to the estimator node by the sensor nodes. *w*(*t*) is the process noise with covariance *Q*, and *v*(*t*) is the measurement noise with covariance *R*. We assume that *w*(*t*) and *v*(*t*) are uncorrelated, zero mean white Gaussian random processes.

**Figure 1.**
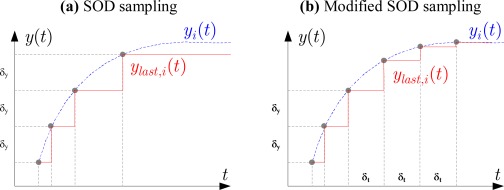
Principle of SOD and modified SOD sampling schemes.

The modified SOD sampling scheme illustrated in Figure 1b is stated as follows:

Let *y_last,i_* (1 ≤ *i* ≤ *p*) be the last transmitted value of the *i*-th sensor output at instant *t_last,i_*. A new sensor value will be sent to the estimator node if one of two following conditions is satisfied:

(2a) $$\left|y_{i}(t)-y_{\mathrm{last},i}\right|>\delta_{y,i}$$

(2b) $$t-t_{\mathrm{last},i}>\delta_{t,i}$$

where *δ_y,i_*, *δ_t,i_* are the given magnitude, time threshold values respectively at the *i*-th sensor node.

Using the modified SOD sampling scheme above we will obtain some benefits. Firstly, the estimator can detect signal oscillations or steady-state error if the difference of output value remains within the threshold range during a long time. Secondly, the estimator can detect multiple packet dropouts if it does not receive sensor data within the interval (0, *δ_t,i_*). Thirdly, theoretical analysis for SOD sampling is still applied for the modified SOD sampling.

However, this scheme has one disadvantage that sensor data transmission rate will be increased due to condition (2b). If *δ_t,i_* is small, the estimator detects packet dropouts fast but data transmission rate is increased. If *δ_t,i_* is large, transmission rate is small but the estimator detects packet dropouts slowly. Therefore, an optimal *δ_t,i_* value is necessary to compromise these constraints.

### 2.1. Multiple packet dropouts detection

The estimator node detects packet dropouts of *i*-th sensor data by checking the instant *i*-th sensor data arrive. If there is no *i*-th sensor data arriving, the estimator node for the time *t* – *t_last,i_* > *δ_t,i_*, then the estimator node knows that one-packet dropout happened at the *i*-th sensor node. Similarly, if there is no *i*-th sensor data arriving for *t* – *t_last,i_* > 2*δ_t,i_*, then two-consecutive-packet dropout happened. We state the general case for multiple packet dropouts as follows:

*If the estimator node does not receive i-th sensor data for time* (*t – t_last,i_*) > *d_i_δ_t,i_* (*d_i_* = 1,2,3,*…*) *then the estimator knows that there have been at least d consecutive packet dropouts at the i-th sensor node since the time receiving y_last,i_*.

**Figure 2.**
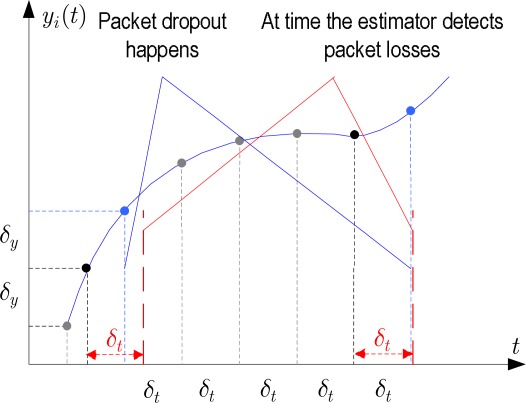
Multiple packet dropout detection.

Note that the estimator just detects “at least” *d_i_* consecutive packet dropouts, not precise *d_i_* consecutive packet dropouts because there exists a delay interval in detecting packet dropouts. As illustrated in Figure 2, although packet loss happens within the time range (*t_last,i_*, *t_last,i_* + *δ_t,i_*), the estimator only detects it at a time (*t_last,i_* + *δ_t,i_*). Thus, if there is more than one packet dropout within the time range (*t_last,i_*, *t_last,i_* + *δ_t,i_*), the estimator also detects only one packet dropout at time (*t_last,i_* + *δ_t,i_*). This is an inevitable flaw of the modified SOD sampling scheme. We can constraint this flaw by reducing the *δ_t,i_* value, but sensor data transmission rate will be increased. Therefore, an optimal *δ_t,i_* value is necessary to compromise between the two constraints.

## 3. State Estimation with Modified SOD Transmission Method

The networked estimation problem applying modified SOD transmission method can be described as follows:

1. Measurement output *y_i_* (1 ≤ *i* ≤ *p*) are sampled at the period *T* but their data are only sent to the estimator node when (2a) or (2b) is satisfied.
2. For simplicity in the problem formulation, transmission delay from the sensor nodes to the estimator node is ignored.
3. The estimator node estimates states of the plant regularly at the period *T* regardless of whether or not sensor data arrive. If there is no *i*-th sensor data received for (*t* – *t_last,i_*) > *d_i_δ_t,i_*, the estimator node considers that the measurement value of the *i*-th sensor output *y_i_*(*t*) is still equal to *y_last,i_* but the measurement noise increases from *v_i_*(*t*) to *v_n,i_*(*t*) = *v_i_*(*t*) + Δ*_i_*(*t*, *t_last,i_*).

Note that if *d_i_* = 0 then there is no packet dropout, the estimator acts like a conventional SOD filter [5]. To formulate a state estimation problem, the boundry of Δ*_i_*(*t*, *t_last,i_*) needs to be determined as *d_i_* ≠ 0 (packet dropouts happen). In the next section, we will compute the covariance of *v_n,i_*(*t*) when *d_i_* ≠ 0 and then a modified Kalman filter is applied for state estimation.

### 3.1. Measurement noise increased due to multiple packet dropouts

We know from (2a) that |*y_i_*(*t*) – *y_last,i_*| ≤ *δ_y,i_* as long as the estimator node does not receive a new *i*-th sensor data value. If one packet dropout happens, the *i*-th sensor output value has changed more than *δ_y,i_*. The estimator should know that:

$$\left|y_{i}(t)-y_{\mathrm{last},i}\right|\leq \delta_{y,i}+\delta_{y,i}$$

**Figure 3.**
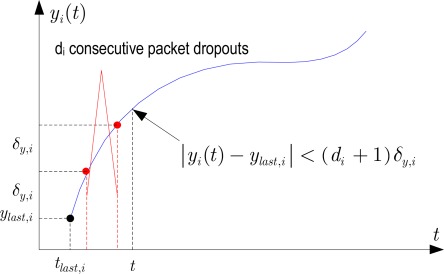
Measurement noise increased due to multiple packet dropouts.

For general cases, as shown in Figure 3, if there are *d_i_* consecutive packet dropouts then:

(3) $$\Delta_{i}(t,t_{\mathrm{last},i})=\left|y_{i}(t)-y_{\mathrm{last},i}\right|\leq (d_{i}+1)\delta_{y,i}.$$

Note that (3) is also applied to the case of no packet dropout [5] by letting *d_i_* = 0. Assuming that Δ*_i_*(*t*, *t_last,i_*) has a uniform distribution with (3), variance of Δ*_i_*(*t*, *t_last,i_*) will be:

(4) $$\begin{array}{lll} E\left[\Delta_{i}(t,t_{\mathrm{last},i})\right] & = & 0\\ E\left[\Delta_{i}^{2}(t,t_{\mathrm{last},i})\right] & = & {\left((d_{i}+1)\delta_{y,i}\right)}^{2}/3\\ \mathrm{Var}\left[\Delta_{i}(t,t_{\mathrm{last},i})\right] & = & E\left[\Delta_{i}^{2}(t,t_{\mathrm{last},i})\right]-E^{2}\left[\Delta_{i}(t,t_{\mathrm{last},i})\right]\\ & = & {\left((d_{i}+1)\delta_{y,i}\right)}^{2}/3 \end{array}$$

Therefore, if there is no *i*-th sensor data received for *t* > *t_last,i_*, variance of measurement noise is increased from *R*(*i,i*) to *R*(*i,i*) + ((*d_i_* + 1)*δ_y,i_*)^2^/3.

### 3.2. State estimation

**Figure 4.**
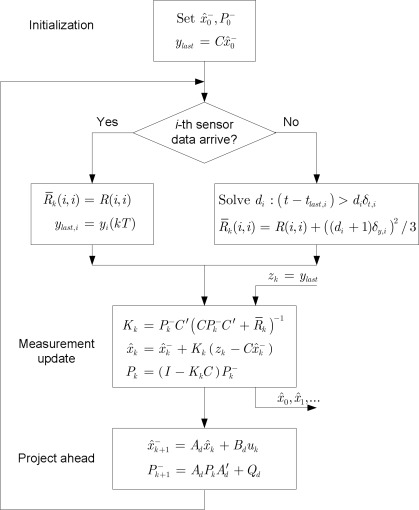
Structure of the modified Kalman filter.

A modified Kalman filter for state estimation *x̂_k_* at step *k*, where there is a change in the measurement update part of the discrete Kalman filter algorithm [15], is given as in the Figure 4. We use the discretized system model sampled at period *T*:

$$A_{d}=e^{\mathrm{AT}},\;\;\;B_{d}=\int_{0}^{T}e^{\mathrm{Ar}}\mathrm{Bdr},$$

where *Q_d_* is the process noise covariance of the discretized system:

$$Q_{d}=\int_{0}^{T}e^{\mathrm{Ar}}{\mathrm{Qe}}^{A^{\prime }r}\mathrm{dr},$$

and *y_last_* is the vector of *p* last received sensor values:

$$y_{\mathrm{last}}={\left[\begin{array}{cccc} y_{\mathrm{last},1} & y_{\mathrm{last},2} & ... & y_{\mathrm{last},p} \end{array}\right]}^{\prime }.$$

In the modified Kalman filter in Figure 4, the states of the plant are estimated regularly at every period *T*, regardless of whether or not sensor data arrive. If *i*-th sensor data arrive then Δ*_i_*(*t*, *t_last,i_*) = 0, the modified Kalman filter acts like the conventional Kalman filter. Otherwise, if *i*-th sensor data do not arrive due to packet loss, it uses *y_last,i_* as the measurement value and *R̄*(*i,i*) = *R*(*i,i*) + ((*d_i_* + 1)*δ_y,i_*)^2^/3 as measurement noise covariance for state estimation.

As stated in [8], if the system (1) is unstable and a packet loss rate is high, the proposed filter could diverge. For example, if all packets are lost, *d_i_* will increase and thus *R̄_i_* will become infinite. Thus *P* in Figure 4 could become infinite.

## 4. Optimal *δ_t,i_* Computing Problem

As mentioned in Section 3, *δ_t,i_* is a trade-off parameter between sensor data transmission rate and the response of packet dropouts detection. The response of packet dropout detection guarantees estimation performance. Because SOD sampling is more efficient than the time-triggered one in network bandwidth improvement, we should choose *δ_t,i_* such that sensor data transmission rate is reduced to promote ability of SOD sampling. In the next section, we will investigate the relation of *δ_t,i_* with transmission rate and the effect of *δ_t,i_* on estimation performance. Then an optimization problem is formulated to find the optimal *δ_t,i_* value according to the given estimation performance.

### 4.1. Sensor data transmission rate by condition (2b)

The total sensor data transmission rate caused by condition (2b) in a time unit:

(5) $$f\left(\delta_{t,i}\right)≜\sum_{i=1}^{p}\frac{1}{\delta_{t,i}}$$

where *p* is the number of sensor output

### 4.2. Estimation error covariance due to packet dropouts

Let *ξ_i_* (0 ≤ *ξ_i_* < 1) be the packet loss rate at the *i*-th sensor node, *ξ_i_* = 0 corresponds to no packet loss. Let Δ*T_i_* be the average transmitting time per packet of the *i*-th sensor node in the conventional SOD method. Note that Δ*T_i_* is dependent on the given *δ_y,i_* value, but independent on *δ_t,i_* value. Δ*T_i_* is computed by running the simulation model in analysis. In practice, it can be computed by letting *δ_t,i_* = ∞ and monitoring the number of packets in a time unit.

The average number of packet dropouts in the conventional SOD sampling per a time unit:

(6) $${\bar{d}}_{i}≜\frac{\xi_{i}}{{\Delta T}_{i}}$$

In the proposed SOD sampling, the average number of packet dropouts within the time interval *δ_t,i_* will be:

(7) $${\bar{d}}_{i}=\frac{\delta_{t,i}\xi_{i}}{\Delta T_{i}}$$

We know from Section 4.1 that the larger number of consecutive packet dropouts is, the larger measurement noise covariance is. Measurement noise covariance is largest if *d̄_i_* packets are consecutively lost. Following the idea in (4), if there is *d̄_i_* packet loss, the measurement noise covariance should be increased as follows:

(8) $$\begin{array}{cc} {\bar{R}}_{\left(i,i\right)} & =R_{\left(i,i\right)}+{\left(({\bar{d}}_{i}+1)\delta_{y,i}^{}\right)}^{2}/3\\ & =R_{\left(i,i\right)}+{\left(\frac{\delta_{t,i}\xi_{i}}{\Delta T_{i}}+1\right)}^{2}\delta_{y,i}^{2}/3 \end{array}$$

### 4.3. Optimal *δ_t,i_* computation

In this section, *δ_t,i_* value is computed. Using (8), we assume that the measurement noise covariance is given by:

(9) $$\bar{R}=R+\mathrm{Diag}\left({\left(\frac{\delta_{t,1}\xi_{1}}{\Delta T_{1}}+1\right)}^{2}\delta_{y,1}^{2}/3,...,{\left(\frac{\delta_{t,p}\xi_{p}}{\Delta T_{p}}+1\right)}^{2}\delta_{y,p}^{2}/3\right)$$

The estimation performance in this case can be computed from the following discrete algebraic Riccati equation:

(10) $$P=A_{d}{\mathrm{PA}}_{d}^{\prime }+Q_{d}-A_{d}{\mathrm{PC}}^{\prime }{\left({\mathrm{CPC}}^{\prime }+\bar{R}\right)}^{-1}{\mathrm{CPA}}_{d}^{\prime }$$

Note that (10) does not provide the actual estimation error covariance of the filter. The main purpose of (10) is to evaluate how *δ_t,i_* affects the estimation performance. We can see that if *δ_t,i_* is large, the estimation error covariance *P* increases.

The solution of (10) is denoted by *P*(*δ_t,i_*). In the following optimization algorithm to find *δ_i_*, we try to reduce the sensor transmission rate caused by condition (2b) subject to the given estimation performance constraint:

*δ_t_* Optimization Problem

(11) $$\begin{array}{l} {\text{min}}_{\delta_{t,i}}\;f\left(\delta_{t,i}\right)\\ \text{subject to}\;\mathrm{DiagP}\left(\delta_{t,i}\right)\leq \mu P_{0} \end{array}$$

where *P*_0_ is the upper bound error covariance with given value *δ_y,i_* and no packet dropout (solution of (10) as *d̄* = *Diag*(0,…,0)). *P*_0_ is also the estimation performance of the conventional SOD. *μ* is the ratio to the estimation performance of conventional SOD filter in case of no packet dropout. If *μ* is large, the *δ_t,i_* optimization problem (11) is done with weaker estimation performance constraints.

## 5. Simulation

To verify the proposed filter, we consider an example of the second-order system with step input where the output is sampled by the SOD and modified SOD sampling:

$$\begin{array}{l} \dot{x}(t)=\left[\begin{array}{cc} 0 & 1\\ -1/a & -b/a \end{array}\right]x(t)+\left[\begin{array}{c} 0\\ M/a \end{array}\right]\;u(t)+w(t)\\ y(t)=\left[\begin{array}{cc} 1 & 0 \end{array}\right]\;x(t)+v(t)\\ Q=0.01,\;R=0.01,\;T=10\mathrm{ms} \end{array}$$

where the system parameters for performance evaluation are given by *M* = 30, a = 5, b = 1 (underdamped system). The simulation process is implemented for 50 seconds.

**Figure 5.**
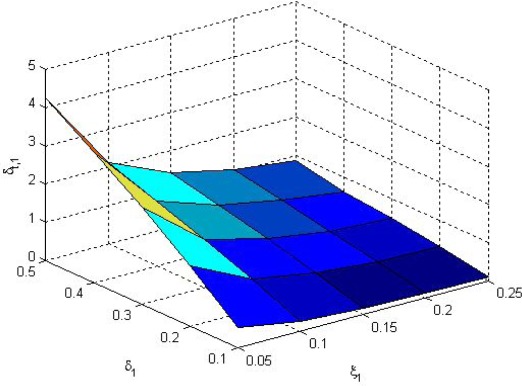
*δ*_*t*,1_ of (11) along with *δ*_*y*,1_ and *ξ*_1_.

**Figure 6.**
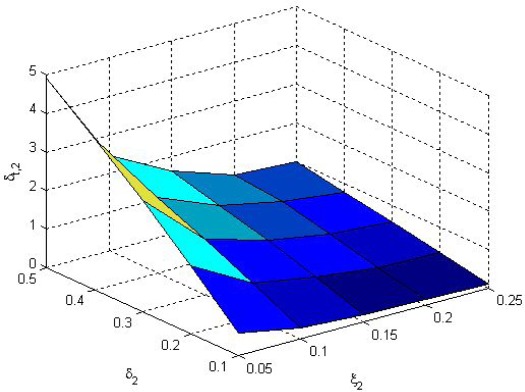
*δ*_*t*,2_ of (11) along with *δ*_*y*,2_ and *ξ*_2_.

Choose *μ* = 5 for the optimization problem (11). The solution *δ*_*t*,1_, *δ*_*t*,2_ of (11) along with *δ_y,i_* and *ξ_i_* are shown in Figures 5 and 6, respectively. We see that *δ_t,i_* is proportional to *δ_y,i_* and reversely proportional to *ξ_i_*. It means that when *δ_y,i_* is large, the *i*-th sensor data transmission rate is small, thus *δ_t,i_* is also small to keep the overall transmission rate small. But if packet dropouts increase (*ξ_i_* is large), *δ_t,i_* value is lowered. As the result, the overall sensor data transmission rate is increased to guarantee estimation performance.

Table 1 shows the estimation error in two filters (SOD filter and modified SOD filter) as *δ*_*y*,1_ = *δ*_*y*,2_ = 0.5, *μ* = 5 and *ξ*_1_, *ξ*_2_ are varying 5%, 10%, 15%, 20%. Estimation error is evaluated by:

(12) $$e_{i}=\frac{1}{N}\sum_{k=1}^{N}{\left(x_{k,i}-{\hat{x}}_{k,i}\right)}^{2}$$

where *x_i_* is the reference state, *x̂_i_* is the estimated state, and *N* = 5,000.

In Table 1, we see that when applying the modified SOD filter, the estimation error is significantly improved. For instance, in the case *ξ*_1_ = *ξ*_2_ = 0.05, the total number of sensor data transmissions in the modified SOD (# 137) is just slightly greater than that in conventional SOD (# 126) but the estimation error is reduced so much ((e_1_ = 0.0075, e_2_ = 0.0096) compared to (e_1_ = 0.0383, e_2_ = 0.0167)).

**Figure 7.**
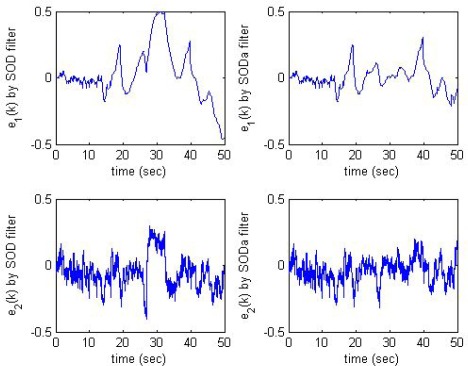
Estimation error in two filters as *ξ*_1_ = *ξ*_2_ = 0.05.

**Figure 8.**
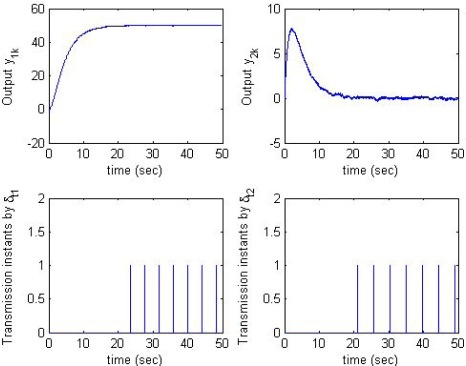
Instants the sensor node transmits data due to condition (2b).

Figure 7 intuitively shows the estimation error in two filters as *ξ*_1_ = *ξ*_2_ = 0.05, *δ*_*y*,1_ = *δ*_*y*,2_ = 0.5, *δ*_*t*,1_ = 4.12, *δ*_*t*,2_ = 4.69. The boundry of *e*_1_ in the modified SOD filter (SODa) is much smaller than that in the conventional SOD filter. Figure 8 shows the instants the sensor node transmits data to the estimator node due to condition (2b). We see that the number of sensor data transmissions caused by condition (2b) is very small in comparison with the total number of sensor data transmissions [(n_1_ = 7, n_2_ = 7) compared to (n_1_ = 101, n_2_ = 36)]. When the modified SOD sampling is applied, the total number of sensor data transmissions is slightly increased, but the estimation error is significantly reduced. Therefore, the modified SOD sampling significantly improves estimation performance with only a little increase in the data transmission rate.

Notice that if we just consider the transmission condition (Equation 2a), estimation error of the proposed method is worse for systems that the output varies slowly. However, an issue of conventional event-based sampling is that it can not detect signal oscillations or steady-state error if the difference of output value remains within the threshold range (because the output varies slowly). This fact causes estimation error to be increased. Whereas, the proposed method uses the transmission condition (Equation 2b) not only to detect packet dropouts but to reduce the error in case the output changes slowly.

As illustrated in Figures 7 and 8, where the estimation error of the proposed method (top-right graph of Figure 7) and of the conventional method (top-left graph of Figure 7) are shown according to the output y1 (top-left graph of Figure 8). We see when y1 varies slowly (time interval from 20s to 50s), the proposed method gives much smaller estimation error than the conventional one.

In case the output changes fast, it is obvious that ignoring packet dropout will introduce extremely incorrect result because we still use the wrong old measurement noise value even when we do not know how much the output value changes.

## 5. Conclusions

In this paper, the state estimation problem with modified SOD transmission method over networks, in which an event-based sampling is combined with a time-triggered sampling to detect packet loss situations, has been considered. We have shown that when using the proposed modified SOD filter, estimation performance is significantly improved with a small increase in sensor data transmission. If multiple packet dropouts happen, the estimator node will detect and compensate for them with an amount of additive measurement noise to improve estimation performance. This method is very useful for networks where data transmission is unreliable due to noise.

## Figures and Tables

**Table 1.** Estimation error along with packet loss rate in two filters.

**Packet loss rate***ξ*_1_ = *ξ*_2_	**0.05(5%)**	**0.1(10%)**	**0.15(15%)**	**0.2(20%)**
n (SOD)	n_1_ = 95			
	n_2_ = 31			
*δ_t,i_*	*δ*_*t*,1_ = 4.12	*δ*_*t*,1_ = 2.08	*δ*_*t*,1_ = 1.73	*δ*_*t*,1_ = 1.52
	*δ*_*t*,2_ = 4.69	*δ*_*t*,2_ = 2.31	*δ*_*t*,2_ = 1.91	*δ*_*t*,2_ = 1.66
n (modified SOD)	n_1_ = 101	n_1_ = 109	n_1_ = 112	n_1_ = 115
	n_2_ = 36	n_2_ = 44	n_2_ = 47	n_2_ = 50
e (SOD)	e_1_ = 0.0383	e_1_ = 0.0384	e_1_ = 0.0386	e_1_ = 0.0391
	e_2_ = 0.0167	e_2_ = 0.0168	e_2_ = 0.0169	e_2_ = 0.0172
e (modified SOD)	e_1_ = 0.0075	e_1_ = 0.0064	e_1_ = 0.0039	e_1_ = 0.0020
	e_2_ = 0.0096	e_2_ = 0.0089	e_2_ = 0.0082	e_2_ = 0.0069
